# Associations Between Fear of COVID-19, Mental Health, and Preventive Behaviours Across Pregnant Women and Husbands: An Actor-Partner Interdependence Modelling

**DOI:** 10.1007/s11469-020-00340-x

**Published:** 2020-06-11

**Authors:** Daniel Kwasi Ahorsu, Vida Imani, Chung-Ying Lin, Toomas Timpka, Anders Broström, John A. Updegraff, Kristofer Årestedt, Mark D. Griffiths, Amir H. Pakpour

**Affiliations:** 1 Department of Rehabilitation Sciences, The Hong Kong Polytechnic University, Hung Hom, Hong Kong; 2 Pediatric Health Research Center, Tabriz University of Medical Sciences, Tabriz, Iran; 3 Department of Health, Medicine, and Caring Sciences, Linköping University, Linköping, Sweden; 4 Department of Nursing, School of Health and Welfare, Jönköping University, Jönköping, Sweden; 5 Department of Psychological Sciences, Kent State University, Kent, OH USA; 6 Faculty of Health and Life Sciences, Linnaeus University, Kalmar, Sweden; 7 The Research Section, Region Kalmar County, Kalmar, Sweden; 8 Psychology Department, International Gaming Research Unit, Nottingham Trent University, Nottingham, NG1 4FQ UK; 9 Social Determinants of Health Research Center, Research Institute for Prevention of Non-Communicable Diseases, Qazvin University of Medical Sciences, Shahid Bahonar Blvd, Qazvin, 3419759811 Iran

**Keywords:** APIM, Depression, Anxiety, Suicidal intention, COVID-19 preventive behaviour, Pregnancy, Dyad

## Abstract

The present cross-sectional study examined the actor-partner interdependence effect of fear of COVID-19 among Iranian pregnant women and their husbands and its association with their mental health and preventive behaviours during the first wave of the COVID-19 pandemic in 2020. A total of 290 pregnant women and their husbands (*N* = 580) were randomly selected from a list of pregnant women in the Iranian Integrated Health System and were invited to respond to psychometric scales assessing fear of COVID-19, depression, anxiety, suicidal intention, mental quality of life, and COVID-19 preventive behaviours. The findings demonstrated significant dyadic relationships between husbands and their pregnant wives' fear of COVID-19, mental health, and preventive behaviours. Pregnant wives’ actor effect of fear of COVID-19 was significantly associated with depression, suicidal intention, mental quality of life, and COVID-19 preventive behaviours but not anxiety. Moreover, a husband actor effect of fear of COVID-19 was significantly associated with depression, anxiety, suicidal intention, mental quality of life, and COVID-19 preventive behaviours. Additionally, there were significant partner effects observed for both the pregnant wives and their husbands concerning all outcomes. The present study used a cross-sectional design and so is unable to determine the mechanism or causal ordering of the effects. Also, the data are mainly based on self-reported measures which have some limitations due to its potential for social desirability and recall biases. Based on the findings, couples may benefit from psychoeducation that focuses on the effect of mental health problems on pregnant women and the foetus.

Mental health problems during pregnancy have been reported to have detrimental consequences on the woman and her foetus (Field 2011; Rees et al. 2019). Depression and anxiety are the most reported mental health problems among pregnant women (Nasreen et al. 2018). The prevalence rate of depression among pregnant women of low- and lower-middle-income countries has been estimated to be 15.6% (Fisher et al. 2012). This usually occurs with other mental health problems and stressors or poor relationship quality with close family members including husbands (Johnson et al. 2018). Although these previous studies mainly focused on pregnant women, few studies have simultaneously explored fathers’ mental health when their wives are pregnant (Nasreen et al. 2018; Wee et al. 2011). Studies have shown that poor couple interaction or poor relationship quality also affects their mental health, such as anxiety or depression symptoms during pregnancy (Figueiredo et al. 2018; Røsand et al. 2012). Therefore, it appears that the behaviour of husbands affects their wives’ mental health and the reverse is equally true (Figueiredo et al. 2018; Røsand et al. 2012). Inasmuch as partners’ negative behaviours affect each other’s mental health, their support or positive behaviour may mitigate their emotional distress or improve their mental health (Ahorsu et al. 2020a; Figueiredo et al. 2018; Røsand et al. 2012).

In addition to the aforementioned personality factors, situational factors such as the novel coronavirus (COVID-19) have also been reported to impact on individual’s mental health. The psychological impact of COVID-19 has focused on populations such as the general population (Wang et al. 2020a) with 58% of the general population rating COVID-19 as having moderate-to-severe psychological impact (16.5%, moderate-to-severe depressive symptoms; 28.8%, moderate-to-severe anxiety symptoms; and 8.1%, moderate-to-severe stress levels). Among medical staff, there was a lower sleep quality which was probably due to their anxiety levels which significantly affected their stress levels and self-efficacy (Xiao et al. 2020). Among pregnant women with COVID-19 infection, studies examining the resultant effect on their foetus and neonates have been inconclusive (Luo and Yin 2020; Yu et al. 2020). However, the clinical characteristics of COVID-19 among pregnant women are similar to non-pregnant adults (Yu et al. 2020) and suggest the existence of psychological burden (Wang et al. 2020a; Xiao et al. 2020). This additional psychological burden may add to the distress of pregnant women especially those with a deficient support system because efficient social support systems may mitigate such problems (Xiao et al. 2020). Therefore, it is appropriate to examine the actor-partner interdependence effect of fear of COVID-19 among pregnant women and their husbands and its association with their mental health and preventive COVID-19 behaviours.

Consequently, in the present study, the Actor-Partner Interdependence Model (APIM) (Fitzpatrick et al. 2016) was used to evaluate the interrelationship between pregnant women and their husbands regarding their fear, mental health, and preventive COVID-19 infection behaviour. The APIM uses actor and partner to represent the interrelated groups (e.g. pregnant women and their husbands in the present study). More specifically, the actor effect is defined as the “extent to which the independent variable of a person influences his or her score on the dependent variable” (Fitzpatrick et al. 2016, p. 75). In the present study, this relates to how much pregnant women or their husbands’ fear is associated with their own mental health and preventive COVID-19 behaviours. The partner effect is defined as the “extent which the independent variable of a person influences the dependent variable of his or her partner” (Fitzpatrick et al. 2016, p. 75). In the present study, this relates to how much pregnant women’s or their husbands’ fear is associated with their husbands or pregnant wives’ (respectively) mental health and preventive COVID-19 behaviours. Moreover, the APIM proposes that there are situations in which two actors are interdependent, and due to this interdependence, it influences each other’s thoughts, emotions, and behaviour, separately from their own (VanderDrift et al. 2017). Therefore, the APIM provides a model for examining dyadic relationships by integrating the concept of interdependence in a two-person relationship (unique associations within- and between-individuals) using appropriate statistical analyses for assessing and testing it (Cook and Kenny 2005; Kenny et al. 2006; VanderDrift et al. 2017). Concerning this study, we used the APIM to examine the dyadic relationship of pregnant women and their husbands’ fear of COVID-19 and its association between mental health, and preventive COVID-19 behaviours. That is, taking into consideration the pandemic nature of COVID-19 and its detrimental effect on people worldwide (Emanuel et al. 2020; Qiu et al. 2020; Wang et al. 2020b), it is appropriate and proactive to assess its effect on this special population (pregnant women) as well as their husbands to investigate a more holistic view of the family’s mental health.

Therefore, the present study examined the interdependencies between fear of COVID-19, mental health, and preventive COVID-19 behaviours among Iranian pregnant women and their husbands during the first wave of the COVID-19 pandemic. The specific objectives were to examine the (i) association between fear of COVID-19 and depression, anxiety, suicidal intention, mental quality of life, and preventive COVID-19 behaviours among pregnant women; (ii) association between fear of COVID-19 and depression, anxiety, suicidal intention, mental quality of life, and preventive COVID-19 behaviours in pregnant women’s husbands; (iii) association between pregnant women’s fear of COVID-19 and their husbands’ depression, anxiety, suicidal intention, mental quality of life, and preventive COVID-19 behaviours; and (iv) association between husbands’ fear of COVID-19 and their pregnant wives’ depression, anxiety, suicidal intention, mental quality of life, and preventive COVID-19 behaviours.

## Methods

### Participants and Procedure

The present study used a cross-sectional design to recruit 290 pregnant women and their husbands who were living in Qazvin, Iran, between March 7 and April 21, 2020 (*N* = 580). The IHS (Integrated Health System: SIB in Persian: http://10.124.253.30/home/login) was used to select pregnant women and their husbands as participants. This system is a comprehensive system for electronic registration of all households in Iran, as well as the registration of all health services that individuals receive from healthcare providers in healthcare centres. In this system, there is full access to the home address and household phone number. Out of 400 pregnant women who were randomly selected from the list of pregnant women registered in the SIB system in Qazvin city (2348 pregnant women), 290 pregnant women and their husbands agreed to participate in this study. Inclusion criteria for the eligible dyads were being (i) at least 18 years old, (ii) able to speak and understand Persian, and (iii) enrolled in the IHS. All pregnant women and husbands read and confirmed the online consent form before completing an online survey. The study procedure was approved by the local Ethical Committee (Qazvin University of Medical Sciences; ref. IR.QUMS.REC.1399.001). An online form which includes the study’s information and questionnaire was designed and sent to the pregnant women and their husbands by SMS. In total, 58% of approached dyads agreed to participate.

### Measures

#### Fear of COVID-19 Scale (FCV-19S)

The FCV-19S, as developed by Ahorsu et al. (2020b), was used to assess participants’ fear of COVID-19. It is a unidimensional seven-item scale. Items are responded to on a five-point Likert-type scale (strongly disagree = 1 to strongly agree = 5). Its total score (summation of individual response items) ranges from 7 to 35 with higher scores indicating greater fear of COVID-19. The Persian version with robust psychometric properties was used (Ahorsu et al. 2020b).

#### Hospital Anxiety and Depression Scale (HADS)

The HADS, originally developed by Zigmond and Snaith (1983), was used to assess symptoms of anxiety and depression levels. The HADS has 14 items divided on two subscales: anxiety and depression. The response format consists of four alternatives (0–3) and the subscale scores (sum of items responded) ranges from 0 to 21. Higher scores indicate higher levels of anxiety and depression. The Persian version with robust psychometric properties was used (Lin and Pakpour 2017; Montazeri et al. 2003).

#### Short-Form Health Survey (SF-12)

The SF-12 (version 2), as developed by Ware et al. (1996), was used to assess health-related quality of life among the participants. It comprises 12 items which cover eight subscales including physical functioning (PF; two items), role limitations due to physical problems (RP; two items), bodily pain (BP; one item), general health (GH; one item), vitality (VT; one item), social functioning (SF; one item), role limitations due to emotional problems (RE; two items), and perceived mental health (MH; two items). In addition to the eight subscales, it includes overall physical (Physical Component Summary, PCS) and mental (Mental Component Summary, MCS) quality of life. The mental health MCS was specifically used to represent mental quality of life in the present study. The Persian version with robust psychometric properties was used (Montazeri et al. 2011; Pakpour et al. 2011).

#### Patient Health Questionnaire (PHQ-9)

The PHQ-9, as developed by Kroenke et al. (2001), was used to assess participants’ depression severity level and more specifically suicidal ideation over the 2-week period prior to the survey. This scale is effective in screening for depression and suicidal ideation due to their strong interrelationship. The scale is a nine-item positively worded questionnaire that is rated on a 4-point Likert-type scale ranging from 0 (not at all) to 3 (nearly every day). Its total score (summation of individual response items) ranges from 0 to 27 with higher scores indicating higher levels of depression. A score above 10 suggests a possible depressive disorder. The Persian version with robust psychometric properties was used (Dadfar et al. 2018).

#### Preventive COVID-19 Behaviour Scale (PCV-19BS)

The PCV-19BS was used to assess COVID-19 preventive behaviours over the past week. It assesses the reported frequency of participation in preventive COVID-19 behaviours as recommended by the World Health Organization (WHO) including washing hands frequently, staying home if feeling unwell, practising respiratory hygiene, and maintaining spatial distancing (World Health Organization 2020a). The PCV-19BS has a Likert scale response format ranging from 1 (strongly disagree) to 5 (strongly agree) which are summed to get its total score. Hence, higher scores indicate greater adherence to engaging in COVID-19 preventive behaviours as recommended by the WHO.

### Data Analysis

Descriptive statistics were performed with mean, standard deviation, number, and percentage values. McNemar’s test, the Friedman test, and paired *t*-test were employed to assess nonparametric and parametric differences between pregnant women and their husbands. Pearson’s correlation coefficient was used to assess the linear relationships between all study variables. Effect sizes of 0.10, 0.30, and 0.50 were considered to be small, medium, and large, respectively (Cohen 1992).

The APIM was used to examine the simultaneous effects of fear of COVID-19 on depression, anxiety, suicidal intention, mental quality of life, and preventive COVID-19 behaviours on the couples (i.e. pregnant women and their husbands). The APIM is a statistical approach that treats a dyad as the unit of analysis (Kenny et al. 2006). The APIM allows the testing of dyadic relationships between couples (i.e. partner effect) while controlling for the relationships between each individual’s scores on independent variable and their dependent variables (i.e. actor effects). The APIMs were conducted utilising a free online app (APIM_SEM) (Stas et al. 2018) using lavaan package in R for fitting structural equation modelling (Rosseel 2012).

To test whether the data were empirically distinguishable, a test of distinguishability was conducted on data using the omnibus chi-square test. Theoretically, the dyadic partners are supposed to be distinguishable (Kenny et al. 2006) based on their genders. A significant chi-square value indicates that actor and partner pathways are different from each other (i.e. distinguishable) (Kenny et al. 2006; Stas et al. 2018). Significant omnibus chi-square values were found for anxiety, depression, suicide ideation, mental quality of life, and preventive COVID-19 behaviours (∆*χ*^2^ (23) = 436.983, *p* < 0.001; ∆*χ*^2^ (23) = 409.011, *p* < 0.001; ∆*χ*^2^ (23) = 418.308, *p* < 0.001; ∆*χ*^2^ (23) = 528.788, *p* < 0.001; ∆*χ*^2^ (23) = 375.762, *p* < 0.001, respectively), indicating that the dyads were statistically distinguished based on the variable of gender. A post-hoc power analysis using APIMPowerR (Ackerman and Kenny 2016) was conducted based on medium effect size (*d* = 0.3) for all actor and partner effects, and the results showed that the minimum required sample size at 80% power was 36 dyads. The Monte Carlo method was used to estimate 95% confidence intervals (CIs) of *k* ratio (the magnitude of the partner effect to the actor effect). A *k* ratio of 1.0 implies that both actor and partners have a relatively equal pattern from the independent variable (i.e. fear of COVID-19) to their dependent variables (i.e. anxiety, depression, suicide ideation, mental quality of life, and preventive COVID-19 behaviours) (Fitzpatrick et al. 2016). All *p*-values of < 0.05 were considered as statistically significant.

## Results

### Demographic Variables

A total of 290 dyads (pregnant women and their husbands) took part in this study. The husbands were significantly older (33.6 years [SD ± 6.4]) and smoked more (*n* = 68; 23.4%) compared with their pregnant wives (29.24 years [SD ± 5.84] and 2 [0.7%], respectively). All other variables such as educational status and substance use were similar between the groups. Approximately one-third of the women (*n* = 87; 30%) were having an unplanned pregnancy, their first child (*n* = 115, 39.7%), and were in about their second trimester (15.04 weeks [SD ± 6]; Table 1).

**Table 1:** Participant characteristics (*N* = 580)

	Pregnant women, mean (SD) or *n* (%)	Husbands, mean ± SD or *n* (%)	*p* value
Age (year)	29.24 (± 5.84)	33.61 (± 6.36)	*p* < 0.001
Educational status			*p* = 0.305
Primary school	40 (13.8%)	50 (17.2%)	
Secondary school	52 (17.9%)	43 (14.8%)	
Diploma	10 (3.4%)	14 (4.8%)	
University	188 (64.8%)	183 (63.1%)	
Currently smoker (yes)	2 (0.7%)	68 (23.4%)	*p* < 0.001
Substance use (yes)	6 (2.1%)	8 (2.8%)	*p* = 0.791
Unplanned pregnancy (yes)	87 (30%)	–	
First child (yes)	115 (39.7%)	–	
Gestational age (weeks)	15.04 (± 6.00)	–	

### Preliminary Analysis

Fear of COVID-19 among pregnant women was significantly and positively associated with their psychological problems (*r* = 0.505 to 0.668; large effect), with their husbands’ psychological problems (*r* = 0.422 to 0.751; medium large effect), with their preventive COVID-19 behaviour (*r* = 0.35; medium effect), and with their husband’s preventive COVID-19 behaviour (*r* = 0.131; small effect). Fear of COVID-19 among pregnant women was significantly and negatively associated with their mental quality of life (*r* = − 0.623; large effect) and with their husbands’ mental quality of life (*r* = − 0.578; large effect) (Table 2).

**Table 2:** Correlation matrix among tested variables

				*r* (*p* value)								
	1	2	3	4	5	6	7	8	9	10	11	12
1. Fear-P^a^	–	0.751**	0.505**	0.621**	0.571**	0.422**	0.668**	0.460**	0.350**	0.131*	− 0.623**	− 0.578**
2. Fear-H^a^		–	0.518**	0.742**	0.771**	0.413**	0.632**	0.481**	0.482**	0.338**	− 0.600**	− 0.653**
3. Depression-P^b^			–	0.409**	0.386**	0.591**	0.353**	0.209**	0.279**	0.097	− 0.605**	0.397**
4. Depression-H^b^				–	0.623**	0.487**	0.605**	0.471**	0.271**	0.258**	− 0.456**	− 0.567**
5. Anxiety-P^b^					–	0.306**	0.596**	0.371**	0.309**	0.259**	− 0.444**	− 0.441**
6. Anxiety-H^b^						–	0.247**	0.111	0.201**	0.094	− 0.454**	− 0.341**
7. Suicide-P^c^							–	0.585**	0.289**	0.267*	− 0.465**	− 0.483**
8. Suicide –H^c^								–	0.224**	0.203**	− 0.297**	− 0.362**
9. Behaviour-P^d^									–	0.204**	− 0.356*	− 0.365**
10. Behaviour–H^d^										–	− 0.050	− 0.226**
11.Mental quality of life–P^e^											–	0.389**
12. Mental quality of life–H^e^												–
Mean (SD)	15.90 (5.29)	17.60 (6.22)	8.06 (3.76)	8.85 (3.67)	7.67 (3.87)	9.98 (4.25)	115^f^ (39.7%)	140^f^ (48.3%)	3.79 (1.27)	3.26 (1.35)	69.67 (15.60)	68.31 (22.46)

^a^Assessed using Fear of COVID-19 Scale

^b^Assessed using Hospital Anxiety and Depression Scale

^c^Assessed using Patient Health Questionnaire Item 9 (PHQ-9)

^d^Assessed using Preventive COVID-19 Behaviours Scale

^e^Assessed using 12-Item Short Form Survey, Mental Component Summary

^f^Any non-zero value of (PHQ)-9 Item 9

*p < 0.05 **p < 0.01

### APIM: Actor and Partner Effects

#### Model 1: Depression

**Fig. 1:**
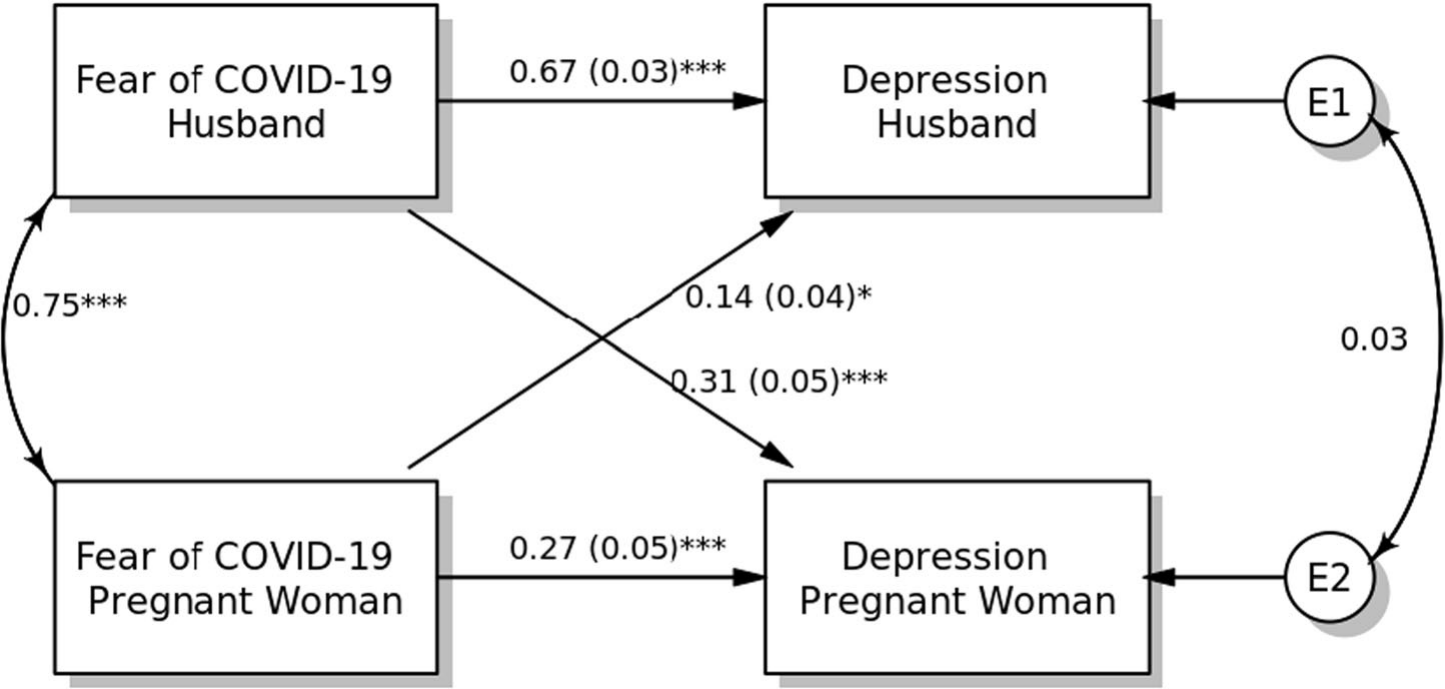
Actor-Partner Interdependence Model of the relation between fear of COVID-19 and depression in pregnant women and their husbands, *p < 0.05; **p < 0.01; ***p < 0.001, *a*(*b*): *β*(SE)

The results of the APIM for depression are shown in Fig. 1. For both pregnant women and their husbands, fear of COVID-19 was significantly associated with their depression level (for pregnant women, *β* = 0.27, SE = 0.05, *p* < 0.001; for husbands, *β* = 0.67, SE = 0.03, *p* < 0.001). Regarding partner effects, fear of COVID-19 was positively associated with higher levels of depression among pregnant women (*β* = 0.14, SE = 0.04, *p* < 0.001) and their husbands (*β* = 0.31, SE = 0.05, *p* < 0.001). The effects of age and smoking status covariates were not statistically significant (*p* > 0.05).

#### Model 2: Anxiety

**Fig. 2:**
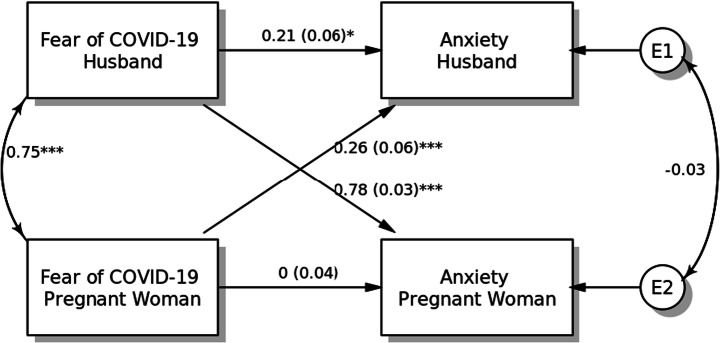
Actor-Partner Interdependence Model of the relation between fear of COVID-19 and anxiety in pregnant women and their husbands, *p < 0.05; **p < 0.01; ***p < 0.001, *a*(*b*): *β*(SE)

Another APIM model examined actor and partner effects of fear of COVID-19 on anxiety (Fig. 2). The fear of COVID-19 among husbands was significantly associated with higher level of anxiety (standardised coefficient [*β*] = 0.21, SE = 0.06, *p* = 0.012). However, no such relationship was observed among pregnant women. Both partner effects were found to be significant when predicting anxiety from fear of COVID-19 among pregnant women (*β* = 0.26, SE = 0.06, *p* < 0.001) and their husbands (*β* = 0.78, SE = 0.03, *p* < 0.001). Moreover, the results of within-dyad covariates for age and smoking status did not show any significant effects on anxiety for either pregnant women or their husbands (*p* > 0.10).

#### Model 3: Suicide Ideation

**Fig. 3:**
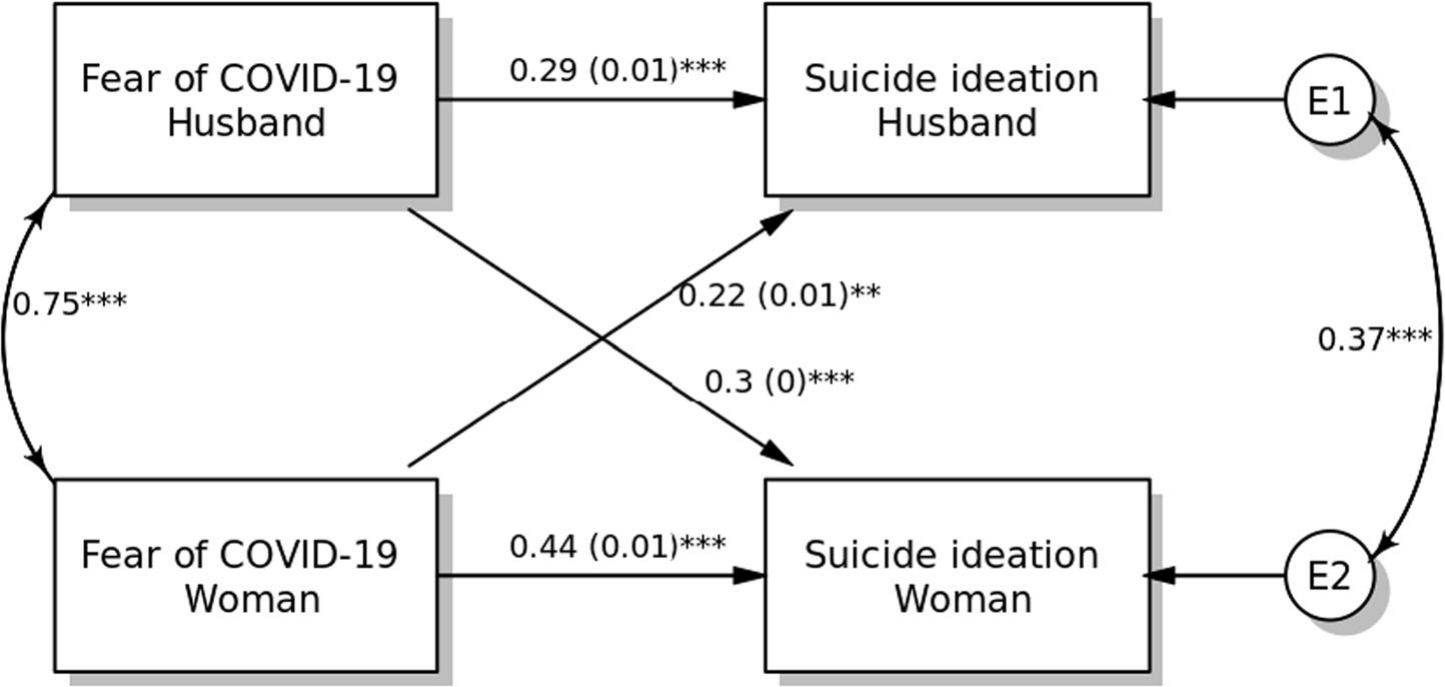
Actor-Partner Interdependence Model of the relation between fear of COVID-19 and suicide ideation in pregnant women and their husbands, *p < 0.05; **p < 0.01; ***p < 0.001, *a*(*b*): *β*(SE)

The APIM model with main actor and partner effects of fear of COVID-19 on suicidal ideation is presented in Fig. 3. Fear of COVID-19 among pregnant women (*β* = 0.44, SE = 0.01, *p* < 0.001) and husbands (*β* = 0.29, SE = 0.01, *p* < 0.001) was positively associated with higher suicidal ideation. Fear of COVID-19 demonstrated partner effects on suicidal ideation for both pregnant women (*β* = 0.22, SE = 0.01, *p* = 0.004) and their husbands (*β* = 0.30, SE = 0.005, *p* < 0.001). Both covariates (age and smoking status) were non-significant in the APIM model.

#### Model 4: Mental Quality of Life

**Fig. 4:**
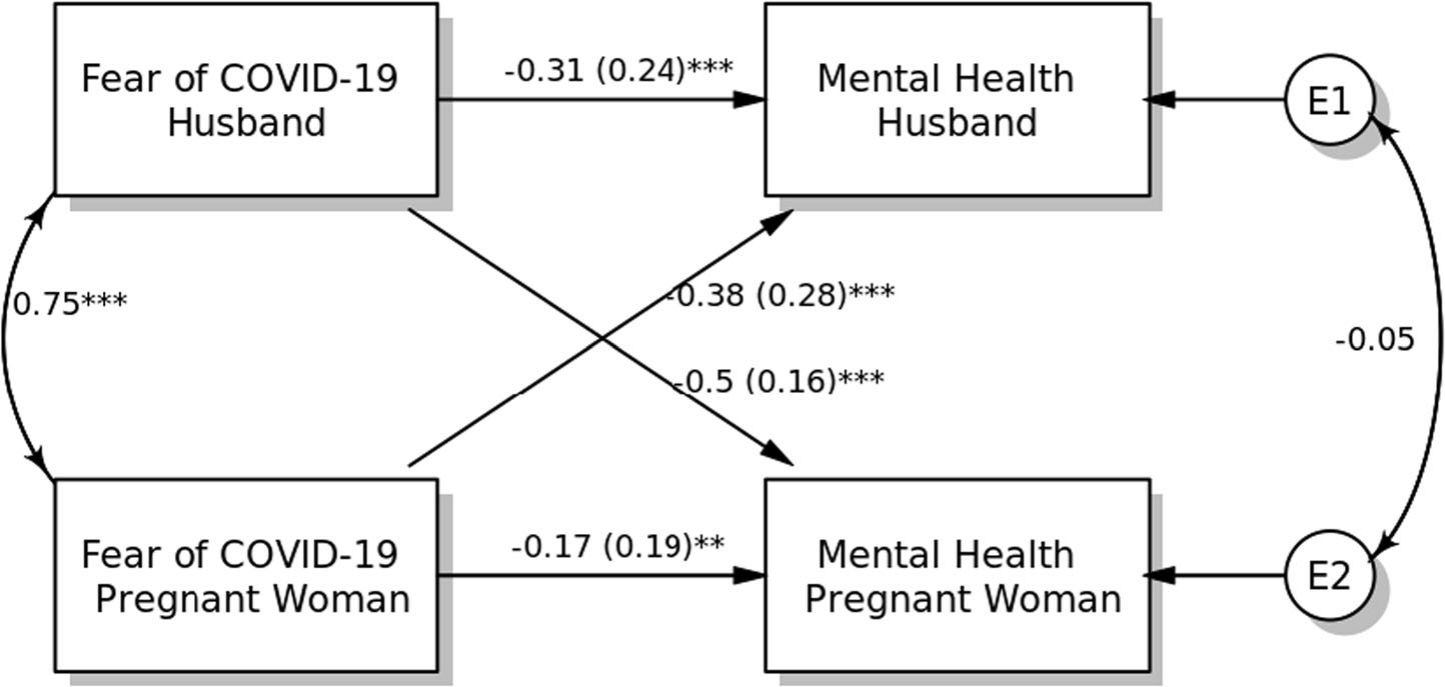
Actor-Partner Interdependence Model of the relation between fear of COVID-19 and mental quality of life in pregnant women and their husbands, *p < 0.05; **p < 0.01; ***p < 0.001, *a*(*b*): *β*(SE)

Fear of COVID-19 demonstrated significantly negative associations with mental quality of life for both pregnant women (*β* = − 0.17, SE = 0.19, *p* < 0.001) and their husbands (*β* = − 0.31, SE = 0.24, *p* < 0.001). Regarding partner effects, pregnant women’s fear of COVID-19 was negatively associated with their husbands’ mental quality of life (*β* = − 0.38, SE = 0.28, *p* < 0.001), and husbands’ fear of COVID-19 was also found to be negatively associated with wives’ mental quality of life (*β* = − 0.50, SE = 0.16, *p* < 0.001). Both within-dyad covariates (i.e. age and smoking status) were not found to be significant in the APIM model (Fig. 4).

#### Model 5: Preventive COVID-19 Behaviour

**Fig. 5:**
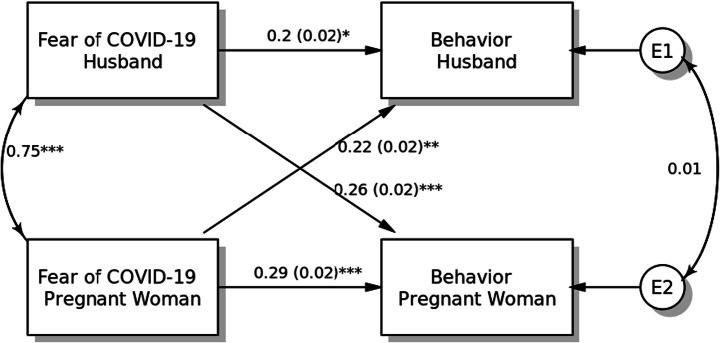
Actor-Partner Interdependence Model of the relation between fear of COVID-19 and preventive COVID-19 behaviours in pregnant women and their husbands, *p < 0.05; **p < 0.01; ***p < 0.001, *a*(*b*): *β*(SE)

The final APIM examined actor and partner effects of fear of COVID-19 on preventive COVID-19 behaviour among pregnant women and their husbands (Fig. 5). The fear of COVID-19 among pregnant women and their husbands was significantly associated with higher preventive COVID-19 behaviours (for pregnant women, *β* = 0.29, SE = 0.02, *p* < 0.001; for husbands, *β* = 0.20, SE = 0.02, *p* = 0.017). Both partner effects were found to be significant when predicting preventive COVID-19 behaviour from fear of COVID-19 among pregnant women (*β* = 0.22, SE = 0.02, *p* = 0.007) and their husbands (*β* = 0.26, SE = 0.02, *p* < 0.001). Moreover, the results of within-dyad covariates for age and smoking status did not show any significance on preventive COVID-19 behaviour for both pregnant women and their husbands (*p* > 0.10).

## Discussion

The present cross-sectional study examined the associations between fear of COVID-19, mental health, and preventive COVID-19 behaviours among pregnant women and their husbands with the dyadic analyses demonstrating noteworthy outcomes. By the time the last data were collected, Iran had approximately 83,500 COVID-19 cases and 5200 deaths (WHO 2020b) after recording their first case a month before in Qom. Therefore, Iranians had adequate information about the pandemic through television, SMS, and online resources with COVID-19 hotlines provided to help Iranians with their queries.

The correlational analyses showed small to large significant relationships between the variables of interest among the pregnant women. These correlational findings were confirmed by the APIM analyses except for the association between fear of COVID-19 and anxiety among the pregnant women. The APIM findings indicated that fear of COVID-19 was significantly and positively associated with depression, suicidal intention, and preventive behaviours but negatively associated with mental health among pregnant women. This suggests that, among pregnant women, higher fear of COVID-19 is associated with higher depression, suicidal intention, and poorer mental health, and vice versa. Fortunately, higher fear of COVID-19 was associated with higher preventive COVID-19 behaviours which suggests that pregnant women had equally instituted procedures to protect themselves from COVID-19 (i.e. its fears). Although significant associations between the mental health disorders such as depression, suicidal ideation, coping strategies, and preventive measures have been well documented (Ahorsu et al. 2020a; Figueiredo et al. 2018; Røsand et al. 2012), these findings add to literature in terms of fear of COVID-19 among pregnant women. These preventive COVID-19 behaviours may be necessary to cope with the stress that comes with COVID-19 because these mental health issues can have inimical consequences to the foetus.

Among the husbands of pregnant women, there were significant correlations between the variables which were supported by the APIM results. The APIM analyses showed that there were significant positive associations between fear of COVID-19 and depression, anxiety, suicidal intention, and preventive behaviours but negative association between fear of COVID-19 and mental quality of life among the pregnant women’s husbands. Similar to the findings of their pregnant women, fear of COVID-19 was associated with other mental health variables including anxiety condition among their husbands. It, thus, seems that fear of COVD-19 should be treated with the same importance as other mental health conditions as they are associated. It is also reassuring to have found a significant association between fear of COVID-19 and preventive behaviours. This would help lessen the impact of these problems and its partner effects on their pregnant wives. Extending previous studies’ findings on the associations between mental health variables and coping, preventing, or supportive strategies (Ahorsu et al. 2020a; Figueiredo et al. 2018; Røsand et al. 2012), this study found that fear of COVID-19 was associated with the aforementioned variables.

The APIM analyses showed that pregnant women’s fear of COVID-19 was significantly and positively associated with their husbands’ depression, anxiety, suicidal intention, and preventive behaviours but negatively with their mental quality of life. This signifies the partner effect of pregnant women’s fear of COVID-19 and its association with the mental health problems of their husbands and their preventive behaviours. Thus, pregnant women’s fear of COVID-19 relates to the mental well-being of their husbands. Hence, couples or partners would have to work together in mitigating the effect of COVID-19 on their mental well-being. This novel finding affirms the interdependence of couples or partners when it comes to the effect of COVID-19 and its association with their mental health.

Similarly, husbands’ fear of COVID-19 was significantly and positively associated with their pregnant wives’ depression, anxiety, suicidal intention, and COVID-19 preventive behaviours but negatively with their mental quality of life. In fact, the partner effects of fear of COVID-19 were substantially stronger for husbands on wives’ anxiety (0.78), mental quality of life (− 0.5), and depression (0.31) than for the partner effects of wives’ fear of COVID-19 on husbands’ anxiety (0.26), mental quality of life (− 0.38), and depression (0.14). This suggests the importance of partner effect of the husbands’ fear of COVID-19 and its significant influence on their pregnant wives’ mental health and preventive behaviours. Consequently, it is important that husbands manage their fear of COVID-19 in order not to add further stress on their wives due to their condition. This finding, although novel in itself, also affirms the view that husbands have significant role to play in the mental health of their wives, especially during pregnancy.

Although greater fear of COVID-19 was associated with poorer mental health, it was related to greater reports of engaging in preventive COVID-19 behaviour. Therefore, in the context of COVID-19, fear appears to promote protective health behaviour. This may be due to the fact that the behaviours of hand-washing and spatial distancing were, at the time of data collection, some of the only few actions that individuals could engage in to prevent COVID-19 exposure and infection. Protection motivation theory (Rogers 1983) posits that when individuals believe they can carry out a behaviour and a behaviour is efficacious against a threat, greater fear should predict greater behaviour. However, given that in the present study fear of COVID-19 was also related to poorer mental health among both the pregnant participants and their partners, research should continue to focus on the effects of fear of COVID-19. One implication of the findings is that efforts to promote hand-washing and spatial distancing should focus more on the efficacy of these behaviours rather than on the threat of COVID-19 infection.

### Limitations

The present study has some limitations. Due to the cross-sectional design of this study, the findings cannot isolate the specific mechanism or causal ordering of the effects. Also, the data are mainly based on self-report measures which have some limitations due to its potential for social desirability bias and recall bias. Nonetheless, these findings can be generalised to all pregnant women and their husbands in Iran due to the sampling strategy used. Also, the study provides novel findings in terms of the dyadic relationship between husbands and their pregnant wives and its association between fear of COVID-19 and mental health, and preventive COVID-19 behaviours.

## Conclusion

The present study demonstrated the dyadic relationships between pregnant wives’ and their husbands’ associations between fear of COVID-19, mental health, and preventive COVID-19 behaviours. The husband’s actor effect of fear of COVID-19 was significantly associated with depression, anxiety, suicidal intention, mental quality of life, and preventive COVID-19 behaviours, while the pregnant wives’ actor effect of fear of COVID-19 was significantly associated with depression, suicidal intention, mental quality of life, and preventive COVID-19 behaviours but not anxiety. Additionally, there were partner effects observed for both the husbands and their pregnant wives in all the outcomes. These findings indicate the mutual effect of fear of COVID-19 of both husbands and their pregnant wives and its association with their mental health and protective COVID-19 behaviours. Consequently, couples are likely to benefit from psychoeducation which focuses on the effect of mental health problems on the pregnant women and the foetus. Future studies should examine the effect of a therapeutic program in mitigating the effect of fear of COVID-19 on the health of pregnant women using the dyadic method.
